# The hidden value of low-performers: ensemble design strategies for coupled ocean-circulation biogeochemical modelling

**DOI:** 10.1038/s41598-026-54424-0

**Published:** 2026-06-08

**Authors:** Ulrike Löptien, Heiner Dietze

**Affiliations:** 1 Department of Computer Science, University of Kiel (CAU), Christian-Albrechts-Platz 4, 24118 Kiel, Germany; 2 Institute of Geosciences, University of Kiel (CAU), Ludewig-Meynstr.10, 24118 Kiel, Germany

**Keywords:** Climate sciences, Environmental sciences, Ocean sciences

## Abstract

Ensemble approaches in weather forecasting and climate science combine the output of several differing models to overcome the limitations of singular models with the idea to exploit the “power of many”. Ensemble members are selected to reflect the full range of the best available knowledge. This often motivates replacing low-performing ensemble members with superior alternatives. Here we propose an alternative approach that benefits from extracting the hidden value of low-performing members in the context of coupled ocean-circulation biogeochemical modelling. We demonstrate for a Baltic Sea coastal test site that, when leveraged with machine learning, a perturbed parameter ensemble of models selected for their capabilities to reproduce extreme dynamics can outperform conventional selection approaches - despite individual rather weak model performance. Implications for assessing global marine biogeochemical projections and ocean geo-engineering options are discussed.

## Introduction

Ensemble approaches combine the output of entire suits of numerical models to overcome the limitations of singular models caused by, e.g., unresolved spatial detail, parametric uncertainties or structural ambiguity^1–4^. Each singular model, also referred to as an ensemble member in this context, is unique in that it differs from the other ensemble members in terms of its characteristic combination of structure, discretisation, numerical methods, parameterisations, initial conditions and forcing. A common approach is to average over the ensemble members with the idea to cancel out individual errors such that predictive performance increases relative to individual deterministic models^5^. At the same time, the ensemble envelope provides some measure for the uncertainties inherent in the predictions. Renown examples for ensemble approaches are the climate projections from the Intergovernmental Panel on Climate Change (IPCC))^6,7^ and weather forecasts^8,9^. Technically, ensemble processing techniques are commonly build upon calculating metrics such as the ensemble mean, the standard deviation or a weighted ensemble mean^10^. When using a weighted ensemble mean, the weights are typically calculated based on statistical relationship between hind-cast simulations and historical observations^11^. More sophisticated state-of-the-art approaches have started to employ machine learning (ML) techniques include skill-based^12^ and regime-based blending techniques^13^.

Ensemble calibration of large ensembles, as common in numerical weather prediction, enhances the reliability further and allows for assigning probabilities for specific events to happen^14,15^.

Compared with atmospheric sciences, ensemble-based techniques in coupled ocean circulation–biogeochemical modelling are in their infancy: so far, the field has largely been restricted to assembling model inter-comparison projects^16^. While this valuable work represents an essential first step by establishing a pool of ensemble members and by providing guidance on their respective uncertainties, efforts to move beyond the ensemble mean and synthesise these models into a single best estimate have not yet been realised. Here we argue that, despite or because of domain-specific challenges, such as especially sparse observations in space and time (compared with, e.g., the global atmospheric observation network), ensemble approaches will mature into the gold-standard for coupled coastal ocean circulation–biogeochemical modelling - simply because there is the need combined with opportunity. The need is grounded in increasing human exposure to coastal hazards which fuels the exploration of management options which, in turn, necessitates reliable forecasting tools in order to simulate what-if scenarios^17–20^. A key difficulty in this context is the assessment of prediction reliability, which becomes particularly important because biogeochemical model equations are not grounded on first-principles, unlike the physical ocean-circulation model components that are essentially based on Newton’s Law. One consequence of this is that many biogeochemical processes are typically parameterised rather than explicitly resolved, which calls for the introduction of a multitude of numerical parameters (like maximum growth rates of phytoplankton or constants describing the relation between growth and ambient temperature). These parameters are typically poorly-known^21–25^ because the diversity and non-linearity of biological systems makes it much harder to constrain them with observations^26–28^ than physical parameters (such as viscosity or Earth’s acceleration). In summary, applications of coupled biogeochemical ocean-circulation suffer from uncertainties^29–32^. The emerging opportunity now is, however, that dramatically cheaper compute makes systematic ensembles of repeated simulations feasible. In this study, we contribute to the resulting question of how best to construct and exploit such ensembles and how to leverage the latter with machine learning. In particular, we explore how to assemble them in terms of perturbation strategy to maximise information gain and constrain uncertainty.

More specifically, we illustrate the benefits of using a perturbed parameter ensemble of coastal coupled ocean-circulation biogeochemical model simulations of the test site Eckernförde Bight in the Baltic Sea. The broader picture here is that this marginal sea in central northern Europe^33^ suffers heavily from anthropogenic eutrophication and related deoxygenation^34–38^. In response to pressing societal needs for robust guidance on eutrophication and coastal management several studies have successfully explored multi-model ensembles of the Baltic Sea^39–41^ and ensembles with differing boundary conditions^42,43^. Outcomes of the respective stake-holder interactions have resulted in explicit quality requirements such as that model results are regraded as acceptable for political decision making when - relative to observations at specific bench-marking observational stations - the model mean bias is less than two standard deviations and the simulated standard deviation does not deviate more than plus or minus 50% from observations^41,44^. Even though these requirements appear moderate, national flagship models still struggle to meet them^45,46^. As a consequence, only very few regional biogeochemical models have ever been operated in forecast mode worldwide^47^. Here we contribute methodological advances by demonstrating practical ensemble-design and ensemble-use workflows that improve the reliability of such model forecasts.

Our test site, Eckernförde Bight, is infamous for intermittent hypoxic events that cause mass fish kill incidents. Further, it is renown for extensive monitoring coverage with one of the longest-operated time series station worldwide^48,49^ at the mouth of the bight and additional monitoring stations such as “Tonne 2a” at the head of bight (9$^{\circ }$52.91’ E, 54$^{\circ }$27.96’ N). This combination of high biogeochemical variability and data coverage has triggered scientific interest in the region ranging from assessing ocean-based geo-engineering^50,51^ to the development of machine learning workflows in biogeochemical forecasting^52^. This study adds to the body of work that has been establishing Eckernförde Bight as a natural laboratory^48–50,52–57^, by testing ensemble-based modelling approaches that offer new avenues for quantifying and predicting complex biogeochemical behaviour.

We use a perturbed parameter ensemble of existing simulations^52^ which simulate dissolved oxygen in Eckernförde Bight. Oxygen has been chosen because bottom concentrations are particularly difficult to simulate while, at the same time, it’s availability governs major biogeochemical processes, including nitrogen removal through denitrification and phosphate release from sediments under reducing conditions. The absence of oxygen is lethal to all higher organisms which has led to the term *dead zones* for regions of hypoxic or anoxic water. Infamous implications of low oxygen concentrations are mass fish kills incidents. Interestingly, dissolved oxygen is among the earliest environmental variables that could be measured with high accuracy, following the establishment of the Winkler method in 1888^58^ which even predates it to standardised salinity measurements introduced by the International Council for the Exploration of the Sea a year later. Hence, there is a relatively large body of oxygen measurements and, consequently, primary sources (atmospheric exchange and photosynthesis) and sinks (microbial respiration) of dissolved oxygen have been confirmed over and over again. Even so, oxygen dynamics remain difficult to simulate quantitatively in both past^59^ and present climates, at global^60^ and regional scales^61^ - mainly because oxygen concentrations at depth are determined by small differences between large and uncertain fluxes (such biotic respiration and physical transport from the surface) integrated over relatively long time-frames^62^.

This study addresses the challenges of oxygen simulation by using an ensemble approach. Our six-member ensemble of simulations (i.e. model versions; Table 1) is designed to capture the major model uncertainties^29,63^ related to the supply and the sinks of oxygen. The model ensemble members differ in terms of vertical background mixing (which is not explicitly resolved because of numerical constraints associated to spatial discretisation) and the parameter settings for local sources and sinks of oxygen, i.e. generation by primary production and consumption at depth and in the sediment. Unlike conventional approaches, that focus on using best-performing models^64–68^ or even only one best model^24,69^, we deliberately include low-performing models with extreme model parameter settings, resembling extreme environmental conditions - such as the absence of all biotic effects (which are highly unlikely to have persisted over the entire simulation period). This model ensemble is then exploited by machine learning to reproduce daily observed subsurface dissolved oxygen concentrations at the head of the bight. Our approach shows a practical alternative to the common practice of selecting one best-performing model by optimising poorly-known biogeochemical model parameters based on observations. Typically such optimization processes require that simulations (or their surrogates^21,63,70^) are rerun hundreds of times to find high-performing simulations which resemble historical or present-state observations. In contrast, we here advocate to apply perturbed parameter ensembles for the desired predictions by mapping out extreme parameter settings and rely on machine learning to decide which combination of simulations is aptly when augmented by suitable (learned) corrections.

**Table 1:** List of model parameter settings for the diffusive background mixing in the MOMBE ocean model of Eckernförde Bight and for the parameters of the EckO$_2$ module: $\kappa$ refers to the vertical background mixing (diffusivity).

Tag	Description	$\kappa$	Opro	Orewa	Orese
		$m^2\,s^{-1}$	$[mmol\,O_2\,m^{-2}\,day^{-1}]$	$[mmol\,O_2\,m^{-3}\,day^{-1}]$	$[mmol\,O_2\,m^{-2}\,day^{-1}]$
LoMix	Low vertical background mixing of momentum and tracers. Local oxygen consumption and production rates at the upper limit of published estimates.	$5 \times 10^{-5}$	48 47 47 46 46 45 48 50 50 49 48 48	3.8 3.8 3.8 3.8 3.8 3.8 3.8 3.8	4 3.5 3 2.5 2.1 1.6 3.95 6.3 5.8 5.4 4.9 4.4
LoMixRem	Low vertical background mixing of momentum and tracers. No local oxygen consumption and production.	$5 \times 10^{-5}$	0 0 0 0 0 0 0 0 0 0 0 0	0 0 0 0 0 0 0 0 0 0 0	0 0 0 0 0 0 0 0 0 0 0 0
MedMix	Medium vertical background mixing of momentum and tracers. Local oxygen consumption and production rates at the upper limit of published estimates	$1 \times 10^{-4}$	48 47 47 46 46 45 48 50 50 49 48 48	3.8 3.8 3.8 3.8 3.8 3.8 3.8 3.8	4 3.5 3 2.5 2.1 1.6 3.95 6.3 5.8 5.4 4.9 4.4
MedMixRem	Medium vertical background mixing of momentum and tracers. No local oxygen consumption and production.	$1 \times 10^{-4}$	0 0 0 0 0 0 0 0 0 0 0 0	0 0 0 0 0 0 0 0 0 0 0	0 0 0 0 0 0 0 0 0 0 0 0
HiMix	High vertical background mixing of momentum and tracers. Local oxygen consumption and production rates at the upper limit of published estimates	$5 \times 10^{-4}$	48 47 47 46 46 45 48 50 50 49 48 48	3.8 3.8 3.8 3.8 3.8 3.8 3.8 3.8	4 3.5 3 2.5 2.1 1.6 3.95 6.3 5.8 5.4 4.9 4.4
HiMixRem	High vertical background mixing of momentum and tracers. No local oxygen consumption and production.	$5 \times 10^{-4}$	0 0 0 0 0 0 0 0 0 0 0 0	0 0 0 0 0 0 0 0 0 0 0	0 0 0 0 0 0 0 0 0 0 0 0

The EckO$_2$-parameters *opro*, *orewa*, and *orese* refer to monthly oxygen production, water column oxygen respiration, and oxygen consumption by the sediment, respectively (one value per month starting with January). Values for *orewa* and *orese* are derived from the published estimates, while *opro* is calculated as residual assuming instant equilibration of sedimentary fluxes. The has been recreated from^52^ and further details are provided here.

## Results

In the following, we illustrate our approach based on a perturbed parameter ensemble of model-based reanalysis of near-bottom dissolved oxygen concentrations at our test sight at the head of Eckernförde Bight. In total our ensemble comprises 6 members (i.e. model versions; Table 1): LoMix, MedMix, HiMix feature low, medium and high background mixing, combined with local biotic sources and sinks of oxygen, respectively. In contrast, LoMixRem, MedMixRem, HiMixRem neglect biotic fluxes entirely (Table 1). From this ensemble we deduce an improved “best-guess” using machine learning. We start with assessing the oxygen concentration at the head of the bay as simulated by individual ensemble members and then evaluate our machine learning approach.

**Fig. 1:**
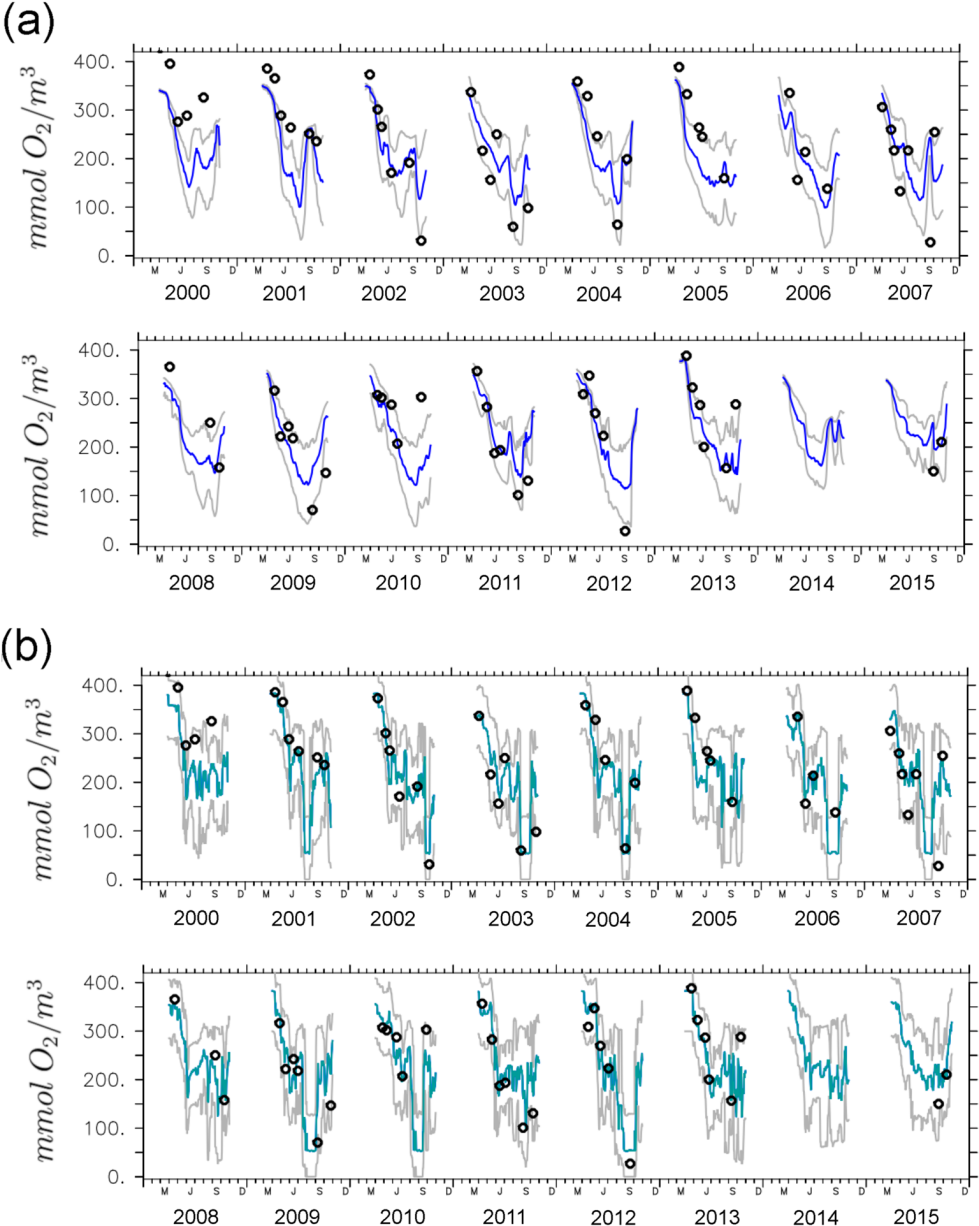
Observed and simulated oxygen concentrations. (**a**) The grey lines depict the ensemble minimum and maximum simulated oxygen values while the blue line shows the ensemble mean. Observations are marked by black circles. (**b**) The grey lines depict the estimated uncertainty range for the ML- prediction (blue-green line). Observations are marked by black circles as above. Units are in *mmol O*$_2$/*m*$^3$. Winter values were not simulated.

*Member’s Performance & Generic Ensemble Approach* Fig. 1 illustrates that Eckernförde Bight is subject to pronounced seasons. Winter is characterised by deep mixing and high oxygen concentrations throughout the entire water column. In spring the surface mixed layer shallows as stratification is building up, driven by air-sea heat fluxes that warm and increase the buoyancy of surface water. This shields deeper layers from air-sea gas exchange and triggers a spring-bloom by increasing the average light experienced by phytoplankton dispersed in the shallowing surface layer^71^ and by affecting the relative effect of grazing pressure by zooplankton^72^. A fraction of the organic material produced by phytoplankton is eventually transported to depth where it is remineralised by bacteria that consume oxygen. The respective oxygen deficit keeps building up until fall when a weakening stratification driven by oceanic heat loss can be overturned by wind-induced mixing associated to strong synoptic weather systems. Superimposed on the local dynamics is the exchange with the greater Kiel Bight^52^. This exchange is, in turn, influenced by intermittent major salt water inflows from the North Sea^73,74^ that replace deep low-oxygenated waters with oxygenated surface water that sinks to depth after passing the shallow Danish Straits. Although all these mechanisms driving the oxygen dynamics have been known since the 19th/20th century, their exact quantitative contributions remains elusive^49^ which maps onto considerable uncertainty in numerical-model based simulations.

The results of our simulations with differing mixing parameterisations are inline with expectations. The model with the highest mixing rate, HiMix, supplies more oxygenated surface water to depth than is the case with reduced mixing in MedMix or LoMix. The simulations LoMixRem, MedMixRem and HiMixRem are respective twins with the difference that in these simulations, all local biotic sinks of oxygen are turned off. To this end the $\star$Rem simulations are extreme cases that assume that all oxygen deficits are imported from outside the model domain rather than being driven by local oxygen consumption.

Despite the wide range of the underlying physical and biological model assumptions the basic dynamics is captured well^52^ by all ensemble members. More specifically we find (Table 2 and Fig. 1): simulated temporal means deviating only between -25% and 10% from the observed mean (i.e. simulated mean values of 180–267 $mmol \ O_2/m^3$ relative to observed 241 $mmol \ O_2/m^3$). Further, set against the benchmarking standards defined for Baltic Sea models^41,44^ we report in Table 2: LoMix is the best-performing simulation with respect to the simulated standard deviation but the mean is biased low such that LoMixRem appears best with respect to the simulated mean and standard deviation. HiMixRem and HiMix are close to poor performing because they underestimate variability. We conclude that the mixing in HiMixRem and HiMix, which smooths out variability, is most likely too strong. Note that this quantitative ranking is a function of how respective criteria are weighted; this inevitably introduces a subjective element to quantitative assessments in general. Other common measures are the Root Mean Square Error (RMSE), which refers to the quadratic mean of the differences between the observed values (i.e. small values are desired) and predicted ones, and the coefficient of determination (R$^2$), which refers to the proportion of explained variance (i.e. large values closest to 1 are desired). For example, MedmixRem scores best regarding the Root Mean Square Error (RMSE) based on the oxygen observations at the observing station “Tonne 2a”. Somewhat surprising, MedMixRem scores generally better than MedMix in terms of RMSE (65.28 $mmol\,O_2\,m^{-3}$ versus 74.26 $mmol\,O_2\,m^{-3}$) and coefficient of determination (R$^2$ score: 0.52 vs. 0.38). One interpretation of this is that the biotic oxygen consumption in MedMix is relatively (too) high because neglecting the consumption (of which we know that it must exist) in MedMixRem produces results that are competitive, based on some common model assessment metrics.

When considering the ensemble mean we find that this generic approach to exploit the “power of many” does not outperform MedMixRem based on R$^2$ score (0.5) and RMSE (66.15). Even when looking closer at the error statistics there is no clear improvement for the ensemble mean relative to best-performing individual members (the 10th and 90th percentile of the errors for MedMixRem are −75.87 and 87.44, compared to −78.23 and 90.36 *mmol O*_2 *m*^-3 for the ensemble mean). Hence, our ensemble is one where the “power of many” apparently fails because the overall variability in the prediction is too low and the errors of individual members do not average out. The latter is not uncommon in biogeochemical ocean modelling and suggests that the errors of the individual members are biased, not independent from one another, or both^75^. To this end, the choice of our ensemble is sub-optimal because the “power of many” fails when considering the ensemble mean. This is, however, a typical situation for coupled biogeochemical ocean circulation model simulations because they are infamous for systematic biases^60,76,77^.

*Accessing the Hidden Value of Low-Performers* Accessing the “power of many” is straightforward when members are unbiased and individual errors are independent from one another. In real-life situations the “power of many” is often easily accessible even if members share biases or correlated errors: imagine a team where all team members tend to miss their deadlines. A skilled manager could compensate for this tendency – for example, by announcing deadlines somewhat too early, depending on the usual time delays of the team members of the past and by learning which team member provides the best estimate depending on circumstances.

Here we follow up this idea by employing machine learning to take the role of a “manager” that learns to derive a best guess based on an ensemble of erroneous, biased and dependent model simulations. More specifically we feed all simulations into a random forest regressor^78,79^ as inputs and with the observations as targets (supervised learning). The random forest regressor fits a number of decision tree regressors on various sub-samples of the dataset and uses averaging to improve the predictive accuracy and control over-fitting. Because, even then, the extremes in our data set were still not captured well we use a quantile regression model to stabilize and regularize extreme predictions in a subsequent step^80^ (see Methods).

**Fig. 2:**
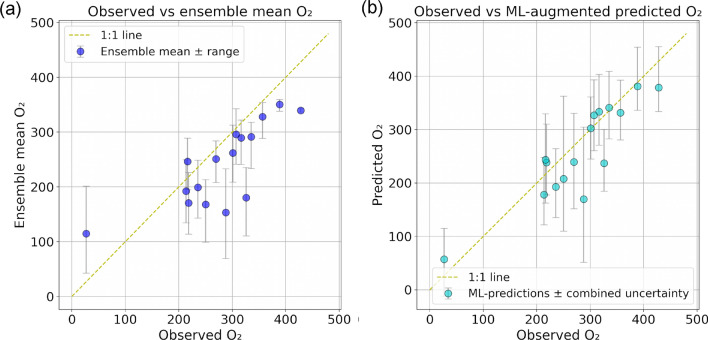
Observed versus predicted values. (**a**) The blue dots show a comparison the ensemble mean to independent test-observations chosen for the ML- prediction. The error bars refer to the ensemble envelope. (**b**) The blue-green dots show a comparison the ML-prediction to independent test-observations (20% random sample of the available observations). The error bars show the square root of the tree-based variability plus the variance of the residuals, while distinguishing between positive and negative values.

By employing this machine learning technique we could substantially enhance the reliability. Our best guess based on an ensemble, including low-performers (e.g. HiMixRem), features an increased R$^2$ score and a substantially-reduced error relative to all individual models and their ensemble mean (Table 3) (R$^2 = 0.74$, RMSE$= 45.8\,mmol \ O_2/m^3$; i.e. $\approx$ 75 % and 30% improvements relative to the ensemble mean). These numbers are based on test data that has not been used in the training. Our approach also provides a measure of uncertainty calculated as the square root of the tree-based 15 and 85%-percentiles (i.e. the variability across trees) plus the variance of the residuals (while distinguishing positive and negative values). This approach has been chosen to capture both, epistemic and aleatoric uncertainties. When tested on independent data (that have not been used to train the regressors), our approach seems reasonable given the good fit and a plausible uncertainty range in Fig. 2. In any case it is superior to a generic baseline approach based simply on the ensemble spread which fails to envelope many observations (as shown in Figs. 1a and 2a). But note that - ideally - RMSE and model spread should match^81 and such a mismatch might indicate model deficiencies^.

Finally, our machine learning approach allows to disentangle the relative value or importance of each ensemble member in putting together the “best guess” (i.e. final prediction) by calculating the so-called Gini-based feature importances^82^. (This is analogous to recording the relative importance assigned to time estimates of certain team members in the management-decision example above). Surprisingly, the relatively low-performing HiMixRem and HiMix contribute substantially more to the final prediction than the much better performing MedMix-models (30 and 20% vs. $\approx$ 13% for both MedMix model versions). Inline with this result and interestingly, taking only the two worst performing and most extreme model versions into account (LoMix and HiMixRem), the prediction is very similar to considering the full ensemble and the RMSE is even slightly improved (Table 3). In contrast, when considering only the two best performing models instead, RMSE and R$^2$ score of the test data worsen compared to using the full ensemble (e.g. R$^2$ score of 0.69 instead of 0.74 obtained for the full ensemble). This result illustrates the hidden value of low-performers in perturbed parameter ensembles and is of relevance in the context of contrary approaches that preference excluding poor performing models from ensembles rather than welcoming diversity^3,83^.

From a statistical point of view we find: When using a Wilcoxon signed-rank test for exploring statistical significance, the difference between the reference and best performing model appears with a p-value of 0.05 on the edge of being significant on the 95%-level. This p-value can be lowered by enhancing the number of trees. As expected, the difference between the reference random forest model and the low performer approach is not significant. Even so, it is remarkable to reach the full performance of the full ensemble with only two (low performing) ensemble members.

In summary: by proposing extreme (but plausible) model parameter settings in a perturbed parameter ensemble, the resulting relatively large model spread provides broad information to the machine learning algorithm which turns out beneficial. These results are inline with insights from numerical weather prediction which propose that forecast probabilities for extreme values should match observed frequencies and perform respective ensemble calibrations^14,15^. To accomplish sufficient diversity for the ML-algorithm, we propose to perform only extreme parameter simulations and leverage these with machine learning in cases where only few model simulations are feasible and parametric uncertainties are large. Another, side effect of using such low performers, resulting from extreme parameter settings, is that these are often less correlated with the observations which can result in lower mutual correlation between the ensemble members, giving the ML algorithm more degrees of freedom. Finally, we find that using extreme model parameters facilitates uncertainty estimation.

**Table 2:** Performance of the ensemble members Mean, standard deviation, coefficient of determination (R$^2$) and root mean squared error (RMSE) for the ensemble members and all observations available in the time period.

Simulation	Mean $mmol\,O_2\,m^{-3}$	Std. $mmol\,O_2\,m^{-3}$	R$^2$ score	RMSE $mmol\,O_2\,m^{-3}$	Performance ranking RMSE	Performance according to earlier studies^41,44^
LoMix	180.20	81.83	0.07	90.99	6	Reasonable/good
LoMixRem	218.41	74.26	0.44	70.64	3	Good/good
MedMix	207.79	73.92	0.38	74.26	4	Good/good
MedMixRem	234.19	68.17	0.52	65.28	1	Good/good
HiMix	253.88	49.08	0.44	70.42	2	Good/reasonable
HiMixRem	267.23	47.04	0.37	74.47	5	Good/reasonable
Ensemble Mean	228.51	64.92	0.50	66.15	(2)	Good/good
Observations	241.86	94.80	–	–	–	–

Note that all model versions differ significantly from the ensemble mean (according to a paired Wilcoxon signed-rank test on test and training data).

**Table 3:** Assessment of the Random Forest Regressor Performance of the random forest regressions on independent random test data.

Model	RMSE $mmol\,O_2\,m^{-3}$	R$^2$ score
Random Forest all	45.7	0.74
Low performer RF	45.5	0.74
Best-off RF	49.8	0.69
Ensemble Mean	67.7	0.42

The “Best-off” RF uses only a subset of the two best performing models according to the RMSE-criterion (Table 2), instead of using all 6 ensemble members as in the first row (i.e. only simulations MedMixRem and HiMix are considered), while the low performer RF focuses on the two worst performing models with extreme parameter settings (i.e. using the simulations LoMix and HiMixRem).

*Application in two Spatial Dimensions* The previous section showed that diverse and apparently low-performing ensembles of numerical simulations of oxygen concentrations could be leveraged with machine learning (a random forest regressor) such that the performance at the monitoring station “Tonne 2a” exceeds the performance of the individual ensemble members and the ensemble mean. Here, we explore if our machine learning approach, that learned on only one observational site, is robust enough to be employed throughout the entire Eckernförde Bight.

**Fig. 3:**
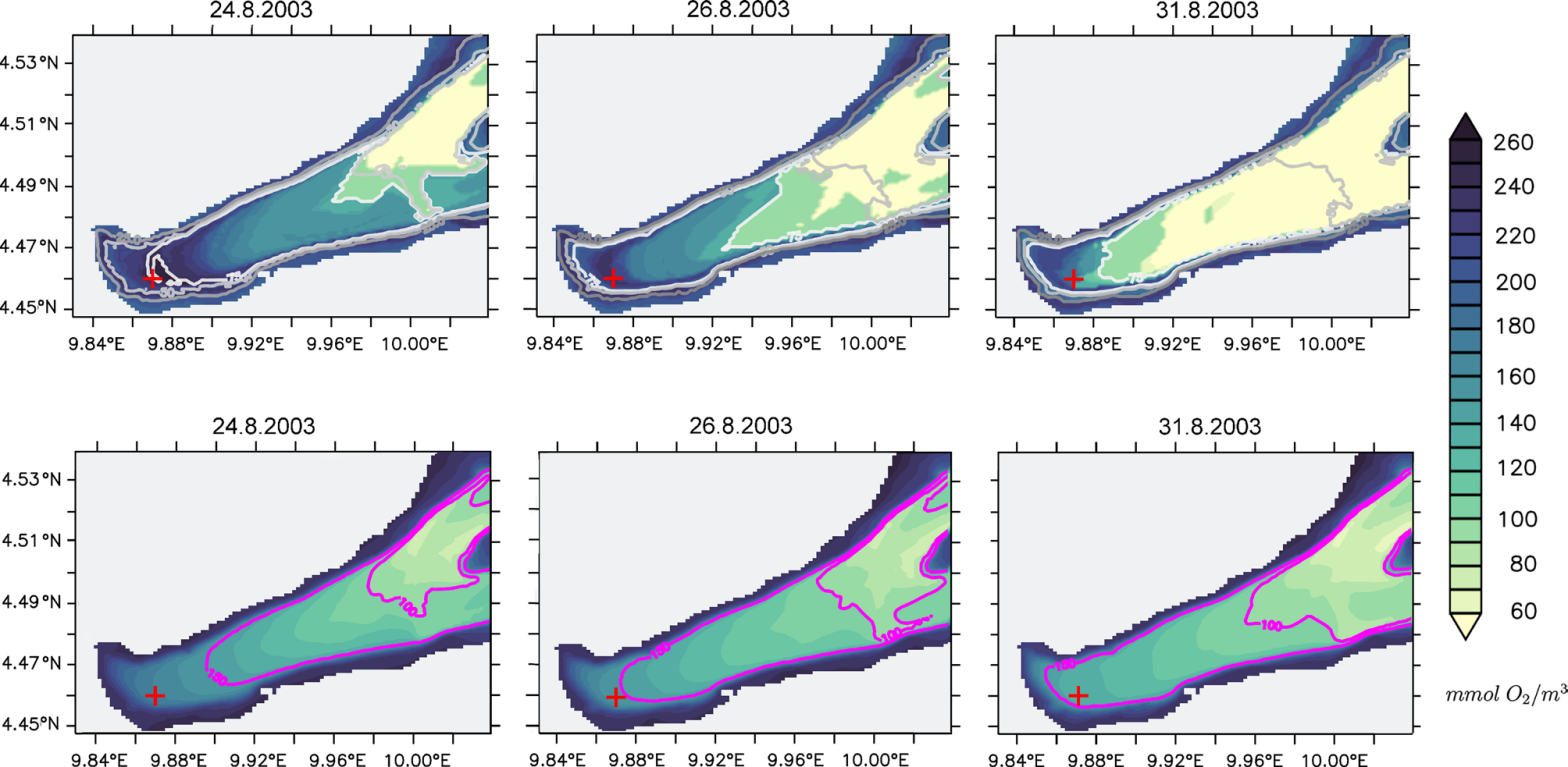
Sanity check of spatial patterns. The upper row shows predicted bottom oxygen concentrations during the arrival of low oxygenated waters in late summer 2003 based on the machine-learning approach. Grey contour lines depict respective error estimates, capturing the levels 25, 50 and 75 *mmol O*$_2$/*m*$^3$. Grey shades decrease such that higher uncertainties are reflected by lighter shades of grey. For simplicity lower and upper errors were averaged. The location of observing station “Tonne 2a” is marked by the red cross. The lower row shows respective ensemble mean simulated oxygen values for comparison. Purple lines show 100 and 150 *mmol O*$_2$/*m*$^3$ isolines.

Technically we make inferences at all horizontal grid points at the deepest (wet) model level, using the ensemble simulations as inputs. This yields time series of 2-dimensional snapshots and provides a sanity test whether the relationships learned from station “Tonne 2a” are applicable to the wider Eckernförde Bight. Fig. 3 (upper row) shows an exemplary hypoxic event in August 2003 as predicted by the ML-based approach. Hypoxic conditions (< 63 *mmol O*$_2$/*m*$^3$^84^) were observed in the deep waters at Boknis Eck at the entrance of the bight on August, 13th. The prediction shows how low oxygenated waters enter Eckernförde Bight, filling the entire bight by the end of the month. Our prediction results are inline with a respective measurement from September, 4th at “Tonne 2a”, showing 63.6 *mmol O*$_2$/*m*$^3$ at depth.

The ensemble mean also shows that relatively low oxygenated waters enter the bight. But, the ensemble mean is less realistic in that it fails to simulate hypoxic conditions at “Tonne 2a”; oxygen levels are considerably higher than observed (Fig. 3; lower row). This is, very likely, related to the too intense vertical mixing in the ensemble members HighMix and HighMixRem.

The ML-inferred bottom-oxygen fields appear as spatially coherent as the ensemble mean and dynamically plausible - which enhances the confidence in the ML approach. In order to account for the enhanced level of uncertainty at those points, where no observations are available, we use a heuristic approach which amplifies the estimated uncertainty range from the reference site by an inflation factor depending on temporal correlations to the reference site and the ensemble spread (see Methods). Resulting mean uncertainty estimates are depicted as contours in Fig. 3. Inline with expectations, the estimated uncertainties are largest in the deep parts of the bight with relatively old bottom waters and much lower for the newly imported low oxygenated waters. Predictions in the shallow waters are estimated to be overall relatively confident which is inline with the fact that the oxygen content of shallow waters is dominated by air-sea gas exchange and errors in biogeochemical source and sink terms do not accumulate so much over time as is the case in deeper waters.

## Discussion

Our study illustrates the potential of machine learning to make best use of an ensemble of coupled biogeochemical ocean model simulations to reproduce vertical profiles of observed oxygen concentrations at a test site in Eckernförde Bight in the Baltic Sea. Even though our ensemble design strategy values diversity over performance by suggesting to use model parameter settings at the edge of the plausible range, a typical problem in coupled ocean-circulation biogeochemical modelling surfaced: despite extreme model parameter settings, the ensemble apparently fails to capture the whole uncertainty range in that it cannot envelope all observations (Fig. 1a). In addition, we have to expect the infamous problem that (at least some) model errors are systematic rather than being independent^60,76,77^ which limits the advantages of using the ensemble mean as “best guess”^75^. In this sub-optimal, but very common situation we find that choosing the ensemble mean for predictions (or hind casts) does not outperform the best performing ensemble member and, further, that by this approach not even the residual error structure is substantially improved.

In contrast, a machine learning-based combination (random forest regression) of the same ensemble members yields markedly better results: A random forest regressor combined with a quantile regressor reduces the RMSE for independent test data considerably from 67.7 for the ensemble mean to 45.7 $mmol\,O_2\,m^{-3}$. Further, the ML-based uncertainty estimates are more reasonable than considering the ensemble spread in that it envelopes more (almost all) observations (Fig.  2b). In this context we have to stress, however, that such a large discrepancy between uncertainty estimate and ensemble spread, despite capturing extreme model parameter settings, suggests some structural model deficiency. Also, we recognise that even our ML-based “best guess” features a considerable error, overall pointing to unresolved processes in our model simulations. Based on a recent study^85^, we speculate that these processes are situated at the interface between the sediment and water column (such as resuspension events) where they cause, often overlooked, events dubbed “hidden seafloor hypoxia”. Another candidate is the representation of vertical mixing which is notoriously difficult to constrain in ocean models also because even just the respective numerical effects are hard to constrain^86–89^.

In summary, we were able to access the hidden value of diverse but individually rather low-performing ensemble members with machine learning. Our approach is in line with concepts for ensemble calibration in numerical weather prediction^14,15^. As for ensemble design, we suggest in the context of biogeochemical modelling, to choose extreme model parameters (within plausible ranges) in order to produce distinct-enough simulations for the ML-algorithm. Following this strategy, even model simulations that performed poorly in isolation contributed meaningfully once their characteristic tendencies were identified (i.e. appropriately regressed by our machine learning approach). This outcome highlights an important implication for ensemble design of biogeochemical projections into our warming future: focusing on high-performant models that most-successfully reproduced observations from the past may fall short when aiming to generate reliable projections and to assess respective uncertainty ranges. A similar conclusion can be drawn when it comes to the evaluation of what–if scenarios, including geo-engineering options. Our results instead suggest that it is advantageous to assemble ensembles that are intentionally diverse, spanning a wide range of parameter settings - even if these would traditionally be discarded due to low individual skill^22,24,25,90–92^. We argue that such diverse ensembles are better suited to capture the entire spectrum of potential system responses and may be leveraged by machine learning for the most informative predictions under present and future conditions.

Caveats remain. Our approach builds on prior knowledge in that, given the observational data, it learns under which circumstances which model is overly optimistic or pessimistic and uses this to calculate a best guess. The problem is that biological systems can abruptly change into entirely different regimes once specific thresholds are exceeded or external triggers come into play^93,94^. Situations like that can mislead the calculation of the “best guess” based on machine learning (irrespective of the underlying numerical algorithms. Finally, this study does not cover structural model uncertainties which may be addressed with multi-model ensembles.

## Methods

### Numerical model simulations and forcing

Our ensemble simulations are based on the ocean-circulation model MOMBE^52^ coupled to the oxygen module EckO$_2$. This coupled model captures the ocean circulation in Eckernförde Bight with an ultra-high resolution of 100m. For comparison, typical state-of-the-art Baltic Sea models have a resolution around 1–3 nautical miles (nm) (i.e. 1.8–5.4 km)^95^ and fail to resolve much of the local circulation in our area of interest.

Our hindcast simulations cover the years 2000–2015. The winter seasons are omitted because winter convection and overall low biotic activity always yield aptly oxygenated conditions. The model has been developed specifically for Eckernförde Bight because of the infamous mass fish kill incidents triggered by a lack of dissolved oxygen^49,52^.

The ocean-circulation model MOMBE is based on the Modular Ocean Model framework MOM4p1, as released by NOAA’s Geophysical Fluid Dynamics Laboratory^96^. Boundaries to the open Baltic Sea are obtained from the observing station Boknis Eck in combination with the Baltic Sea model MOMBA^74,97,98^. Following an earlier approach^99^, the module EckO$_2$ is coupled to the ocean -circulation component. The complexity of EckO$_2$ is kept on a relatively low level to limit the amount of poorly known model parameters and to enable us to span the uncertainty range with few simulations. Among the challenges in simulating oxygen dynamics (as with many other biogeochemical tracers) is that both biotic parameters (determining oxygen respiration) and the antagonistic abiotic parameters (that control ventilation with surface water high in oxygen such as, for example, vertical diffusivity) are relatively uncertain. Our approach to this problem is to run an ensemble of simulations encompassing a plausible range of settings. These settings are listed in Table 1. We compare low, medium, and high levels of diffusivity (tagged LoMix, MedMix, and HiMix, respectively) and, further, simulations which totally neglect local sources and sinks of oxygen (tagged $\star$Rem for “remote biotic effects only”) versus those featuring a best guess of local sources and sinks that are on the higher end of published estimates. Since oxygen levels are generally high in winter these months were not considered. The model setups are published and have been described in detail earlier^52^.

### Random forest regression

The choice of a machine-learning–based post-processing approach is motivated by the comparatively sparse observational data availability, relative e.g. to atmospheric sciences. This sparsity limits the feasibility of training highly complex models, such as deep neural networks, without a substantial risk of overfitting. Consequently, we employ a random forest regression model and deliberately restrict the number of input variables.

Random forest regression^78,79^ is among the most widely used machine learning techniques. A random forest regressor consists of an ensemble of regression decision trees^100^. In contrast to linear regression, decision trees partition the feature space in a hierarchical, rule-based manner, enabling the representation of complex and non-linear relationships between predictors and the target variable. However, individual decision trees are prone to overfitting. This issue is mitigated in a random forest by aggregating multiple trees trained on bootstrapped samples and randomised feature subsets. The final prediction is obtained as the average of the individual tree predictions.

In the present study, we use 80% of the available observations at the observing station “Tonne 2a” to train a random forest regressor that combines the available numerical model outputs (6 ensemble members with daily sampling) in order to reproduce the observations of near bottom dissolved oxygen in Eckernförde Bight as accurately as possible. Variations of this approach refer to the “Low-performer” and “Best-off” random forests which take only two ensemble members into account (cf. Table 3). The random forests consist of 120 trees; increasing this number by a factor of two or three had only a negligible effect on the results. The minimum number of samples required at a leaf node is set to two. The maximum number of features and maximum tree depth were not limited. Due to data sparsity in the lower tail of the prediction the variance among trees is generally high and the extreme predictions contain an optimistic bias. To overcome this problem, we apply a post-prediction correction designed to improve the behaviour to stabilise and regularise prediction in low-value regimes by selectively blending the model’s point prediction with an externally estimated lower quantile prediction (i.e. a quantile regression model^80^) whenever a prediction undercuts the threshold of 90 mmol O$_2$/m$^3$. Specifically, the method computes a convex combination of the original prediction and the 5th percentile estimates (30% original prediction and 70% lower quantile estimate). The problem is less pronounced for the high extremes where more trainings data are available. Still, an analogue procedure is also applied to high extremes above 370 mmol O$_2$/m$^3$ and this could induce another small performance gain. Note that all other, non-extreme predicted values are not affected. Statistical significance is tested using Wilcoxon signed-rank test. It is a nonparametric statistical paired difference test which tests if the median difference between paired samples is zero. Significance indicates that there is a systematic shift between the data. Wilcoxon signed-rank test is a well-known alternative to Student’s t-test if the distribution of differences is not normal (or unknown).

To obtain a measure for the predictive uncertainty we combine tree-based uncertainty from the random forest with residual-based uncertainty, while explicitly distinguishing between positive and negative deviations to allow for asymmetric prediction errors. For the residual-based uncertainty we use local out-of-bag (OOB) residuals to approximate test-set error which approximates how wrong the model is on unseen data. The tree-based uncertainty is estimated using the 15th and 85th percentiles of the individual decision tree predictions, which characterise the spread across trees and reflect uncertainty arising from the stochastic nature of the random forest algorithm. The lower and upper bounds for the total predictive uncertainty is then defined as the square root of the sum of the tree-based variability and the variance of the model residuals (Eq. 2):

1 $$\begin{aligned} uncertainty\_lower = \sqrt{\sigma ^2_{resid-}+P_{15, trees}^{\ 2}} \end{aligned}$$

2 $$\begin{aligned} uncertainty\_upper = \sqrt{\sigma ^2_{resid+}+P_{85, trees}^{\ 2}} \end{aligned}$$

where $P_{15, trees}$ and $P_{85, trees}$ denote the 15 and 85% percentiles of the decision tree predictions, respectively. These percentiles are chosen to approximately correspond to one standard deviation of the tree ensemble. The terms $\sigma ^2_{resid-}$ and $\sigma ^2_{resid+}$ represent the variances of negative and positive residuals, respectively. Including these residual variances account for discrepancies between predictions and observations that are not captured by inter-tree variability alone, particularly those arising from structural uncertainties or systematic errors in the underlying numerical model^101^ (i.e. we aim to capture both, epistemic and aleatoric uncertainties). Note, however, that this approach is heuristic since OOB residuals and variance are not strictly independent.

To test both the uncertainty coverage and the predictions, we use a randomly selected 20% of the observational data which is retained as an independent test set. We used a joint, aligned split on predictors and test data where data indices were shuffled randomly. We do not expect data leakage because observation lie several weeks apart from one another (which exceeds the memory of the system). When the trained random forest model is applied to other locations within the bight where no in situ observations are available, uncertainty estimates are increased accordingly. This is achieved using a pragmatic inflation factor (Eq. 2), derived from a spatial correlation map and the numerical model ensemble spread ratio. The correlation map is constructed from the mean temporal correlations between each ensemble member and the reference site “Tonne 2a”. Higher similarity to the reference site, measured by the absolute correlation, results in a smaller inflation factor. An additional modulation of the inflation factor is based on the ratio of the local numerical model ensemble spread to that at the reference site. Locations with a larger ensemble spread than “Tonne 2a” experience increased inflation, while locations with smaller ensemble spread experience reduced inflation:

3 $$\begin{aligned} inflation = (1 + 0.3 * (1 - |corr|)) *spread\_ratio, \end{aligned}$$

The spread ratio is defined as the local numerical model ensemble spread divided by the ensemble spread at the reference site “Tonne 2a”. By construction, the inflation factor at the observational site “Tonne 2a” equals one. We anticipate that these uncertainty estimates will decrease as additional observations become available in the future.

## Data Availability

Meridional sections and bottom values of simulated oxygen concentrations, temperature, salinity, residence time, and age have been visualised for the hindcast period for a stakeholder. These are archived under https://doi.org/10.5281/zenodo.4271941 (Dietze and Löptien, 2020). Respective data and input files for the model are provided under the link https://doi.org/10.5281/zenodo.18622070.

## Code availability

The circulation model code MOM4p1 is distributed by NOAA’s Geophysical Fluid Dynamics Laboratory (https://github.com/mom-ocean/MOM4p1, last access: 22. Jan. 2026, Griffies, 2009). We use the original code without applying any changes to it. The code for model valuation, random forest generation and plotting routines are provided under the link https://doi.org/10.5281/zenodo.18622070. Fig 2 was created with Python while Figs. 1 and 3 were created with Ferret (NOAA).
